# An investigation of combined effect of infill pattern, density, and layer thickness on mechanical properties of 3D printed ABS by fused filament fabrication

**DOI:** 10.1016/j.heliyon.2023.e16531

**Published:** 2023-05-23

**Authors:** Anant Prakash Agrawal, Virendra Kumar, Jitendra Kumar, Prabhu Paramasivam, Seshathiri Dhanasekaran, Lalta Prasad

**Affiliations:** a Department of Mechanical Engineering, Noida Institute of Engineering and Technology, Greater Noida, 201306, India; b Department of Mechanical Engineering, Harcourt Butler Technical University, Kanpur, 208002, India; c Department of Mechanical Engineering, Mattu University, 318, Ethiopia; d Department of Computer Science, UiT the Arctic University of Norway, Tromso, 9037, Norway; e Department of Mechanical Engineering, National Institute of Technology, Uttarakhand, 246174, India

**Keywords:** ABS, FDM, Fused filament fabrication, Additive manufacturing, Tensile strength, Impact strength

## Abstract

Additive manufacturing technology and its benefits have a significant impact on different industrial applications. The 3D printing technologies help manufacture lightweight intricate geometrical designs with enhanced strengths. The present study investigates the blended effects of previously recommended parameters of different infill patterns (line, triangle, and concentric) and infill densities (75, 80, and 85%) with varying thicknesses of layers (100, 200, and 300 μm). The test samples were created through Fused Filament Fabrication (FFF) technology using Acrylonitrile Butadiene Styrene (ABS) 3D printing. Mechanical properties were evaluated through tensile and impact strength tests conducted in accordance with ASTM standards. The experimental investigation reveals that the infill pattern greatly affected both tensile and impact strength. The best results were obtained with a concentric infill pattern, along with 80% infill density and 100 μm layer thickness. These conditions resulted in 123% and 115% higher tensile strength and 168% and 80% higher impact strength compared to line and triangle patterns, respectively.

## 1 Introduction

An eco-friendly production process design is vital in advanced manufacturing applications. The 3D Printing process is a progressive, unique, original, and innovative additive manufacturing technology. The capabilities of progressive Additive Manufacturing (AM) processes to create free-form shapes without limitations and quickly implement new designs are making them more important than traditional subtractive methods in Industry 4.0 [1]. The most widely implemented AM technique in various engineering and medical applications, including automotive, aerospace, biomedical [2], sports, and civil field, for the speedy creation of functional polymer-based [3] parts is selective material deposition using the hot extrusion process, which is commonly referred to as FFF [4].

**Fig. 1:**
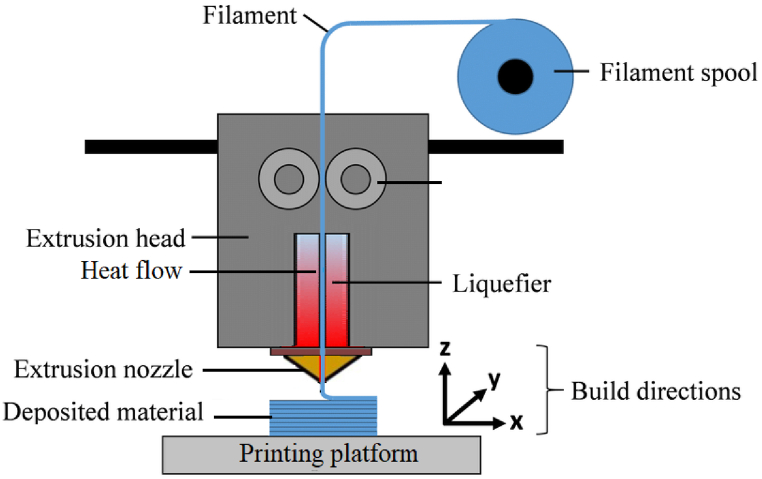
Fused filament fabrication (FFF) schematic diagram.

Rapid prototyping can create a complex geometrical shape through digitation without wasting materials during conventional machining [5]. The techniques include liquid-based methods such as stereolithography (SLA) and solid-based techniques like fused deposition modeling (FDM), selective laser melting (SLM), and selective laser sintering (SLS). Because of its versatility in generating mesostructures, FDM is one of the most effective 3D printing processes. In this technique, raw material is extruded through the nozzle in a semi-liquid state and built required shape layer by layer, A schematic diagram of the FFF process is shown in Fig. 1. The Fused Filament Fabrication (FFF) method in 3D printing allows for the production of parts with varying infill densities and patterns, making it possible to determine the most suitable structure. Many process variables regulate the functional properties of the FFF-manufactured components. Build orientations, infill density [6], width, raster angles, layer thickness, and air gaps are essential in determining mechanical properties.

The polymeric structures are durable and have enhanced high resistance to impact strength with lightweight. The mesostructures (infill density and pattern) play an important role in maintaining an appropriate balance of stress intensity and controlling crack propagation.

Raj S.A. et al. [5] studied the mechanical and biodegradable properties of 3D printed PLA material [6]. They observed that PLA could be better alternative material to ABS. Lay M. et al. [7] evaluated the physical and mechanical characteristics of nylon-6, ABS, and PLA manufactured by injection molding and FDM processes. The study found that FDM (Fused Deposition Modeling) samples have a significantly higher water absorption rate compared to those produced through injection molding, with a difference of approximately 108%. However, the impact strength, percent of elongation, Young's modulus, and tensile strength of FDM samples were weaker, with values that were about 78%, 48%, 50%, and 48% lower respectively compared to the injection molding samples. Shabana R. et al. [8] examined the mechanical characteristics of 3D printed ABS and PLA thermoplastics using surface roughness, microhardness, compressive strength, flexural strength, and tensile strength. The research findings indicate that, among the ABS and PLA thermoplastics, ABS demonstrated greater elongation and flexural strength prior to breaking. On the other hand, PLA was found to have a higher Ultimate Tensile Strength (UTS) compared to ABS. The red-colored PLA had the highest elastic modulus, YS, and UTS, whereas the pink-colored PLA had the most increased toughness and % strain among 13 different colored PLA [9]. In his experimental study, Kannan S. et al. [10] reported that compared to ABS and Polycarbonate (PC), *P*C-ABS material showed enhanced elastic limit and load-bearing performance. Salim M.A. et al. [11] concluded that PLA required higher stress to deformation than ABS because the tensile and flexural strength of PLA was 7% and 9% higher than ABS. Panes A. et al. [12] also observed that PLA had better mechanical performance than ABS in reference to layer height, infill density, and layer orientation.

Build orientation, layer thickness, raster width, raster angle and infill % are process variables that substantially impact the mechanical characteristics of FDM manufactured parts. Previous research used many optimization methods and techniques to optimize these process parameters to enhance the mechanical characteristics of the part. N. Vidakisa et al. [1] examined the impact of printing process parameters on energy consumption in sustainable manufacturing. These parameters were infill density [13], raster angle, nozzle temperature, printing speed, layer thickness, and bed temperature [14]. According to the researchers' findings, an increase in printing speed and layer thickness led to a noteworthy decrease in both Printing consumption and Specific printing energy. Nevertheless, they also observed that such an increase in speed and thickness had an unfavorable impact on mechanical strength, as determined by mechanical strength evaluation tests.

Yao T. et al. [15] firstly theoretically predicted UTS of PLA with different printing orientations from 0 to 90° and three-layer thickness by theories such as classical lamination theory, transverse isotropic hypothesis, and Hill-Tsai anisotropic yield criterion and after that verified it experimentally. This study observed that UTS decreases with smaller printing angles or thicker layers. Mishra P.K. et al. [16] examined how impact strength was affected by various infill patterns and infill densities. The study found that for 85% infill density, impact strength was more due to the dependency on the nature of the mesostructure. The delamination of mesostructure layers breaks the crack propagation continuity during impact.

Milovanovic A. et al. [17] reported that PLA-X material possessed higher mechanical properties than ABS as compared to PLA material at different printing parameters. The process parameter, raster angle, infill percentage, printing speed and layer thickness were optimized by Algarni M. et al. [18] on PETG, PEEK, ABS, and PLA materials using ANOVA. According to the study, the infill density is the key process variable that affects PLA and ABS. As per Nugroho A. et al. [19], when layer thickness increased from 0.4 to 0.5 mm, the flexural strength of 3D printed PLA material significantly increased. Khatwani J. and Srivastava V [20]. investigated how the part bed temperature, the layer thickness, and the nozzle diameter affected the tensile and flexural strength of PLA. The study shows that the flexural and tensile strengths increase with the part bed temperature increment. In contrast, with an increment in the layer thickness, the tensile and flexural strength decreased and increased, respectively.

Moradi M. et al. [21] conducted a study that analyzed the mechanical performance of 3D printed PLA based on different infill patterns. The results showed that, among the various infill patterns, the triangular pattern had the highest Ultimate Tensile Strength (UTS) and Young's modulus. On the other hand, the wiggle and fast honeycomb pattern were found to have the greatest ductility, elongation, and toughness, attributed to their flexible structure. Wu W. et al. [22] reported that the bending strengths, compressive, and tensile and of 3D printed PEEK were 115%, 114%, and 108% and superior to ABS for 300 μm layer thickness and a 0° raster angle. Hanon M.M. et al. [23] and Galeja M. et al. [24] also reported that orientations and raster angle significantly affected mechanical properties. Gunasekaran K.N. et al. [25] results showed that the specimens printed with 100% infill density have improved mechanical properties in terms of flexural strength, impact strength, tensile strength, and hardness [25]. Kam M. et al. And Fernandes J. et al. [26] reported that thickness of layer [27,28] had the most significant impact on the enhancement of mechanical properties instead of extruder temperature, occupancy rate, filling structure [29], and infill pattern [30] whereas, Saini J.S. et al. [31] reported print orientation angle 67.5° played a significant role on the SLA fabricated polymer material's mechanical properties. Algarni M [32]. concluded his research that 10% moisture content and 90° raster angle significantly affected the UTS by increasing 36%.

Vidakis N. et al. [33] examined the effect of different printing parameters on the mechanical strength of ABS [34] and ABS-plus materials under bending and impact conditions. The results of Samykano M. et al.’ study showed that the optimal mechanical properties of ABS using FDM technology were achieved with 65° raster angle, 0.5 mm layer thickness and 80% infill percentage as process parameters [35]. According to Dwiyati S.T. et al. [36] the highest force and tensile strength were seen in the axial direction of 3D printed specimens, compared to the lateral direction. They noted that greater maximum force and tensile strength were present in specimens with thicker layers. Nomani J. et al. [37] discovered that smaller layer thicknesses resulted in higher material strength and stiffness compared to larger layer thicknesses. This was due to the increased number of deposited layers enhancing interlayer bonding strength, as well as the shear hardening effect of the extrusion process. Raja S. et al. [38] reviewed mechanical properties and optimized the FDM printing process parameters for PLA with respect to lowest production time [39].

According to literature, proper selection of manufacturing parameters can result in the production of high-quality parts or components with the desired mechanical properties. Optimal setting of these parameters leads to the production of highly effective parts. The majority of the effort involved changing one or two process parameters to measure the mechanical properties. The subject of a few works listed is the exploration of mechanical properties through the simultaneous change of a number of process parameters (control factors).

From the previous work of various researchers, line [6], triangle [18], and concentric [6] infill patterns, 75–85% infill densities [6,18], and 100–300 μm layer thickness [17,18,20], and found among the suggested best process parameters for the 3D printed components strength. The novelty of this work is to analyze and optimize which one will be the best combination among these suggested best parameters. Therefore, the focus of the present work is to investigate the influence of the FFF process parameter's infill pattern (line, triangle, and concentric), infill density (75%, 80%, and 85%), and layer thickness (0.1 mm, 0.2 mm, and 0.3 mm) on the mechanical properties of parts made up of ABS. The study begins with preparing a sample on a 3D printer using an ABS wire spool based on ASTM standards to investigate the strength of the material. The mechanical properties of the printed samples are assessed through tensile strength and Izod impact strength testing.

## 2 Materials and methods

In this study, ABS material was considered as it has better mechanical properties (*i.e.,* impact strength) [7] than other standard materials, such as PLA and Nylon 6. It offers faster printing rates and more heat resistance; however, the disadvantage of ABS over PLA is that it shrinks during 3D printing, resulting in poor dimensional accuracy or printing failure. In this work a high impact grade (ABS-3D HI) ABS (Acrylonitrile Butadiene Styrene) filament used, manufactured by 3DXTECH an American manufacturer with a diameter of 1.75 mm and a density of 1.05 g/cc.

Fused Deposition Modeling (FDM) is an extrusion-based 3D printing process that creates solid objects by melting thermoplastic materials and extruding them through a nozzle onto a building platform, layer by layer starting from the bottom. The mechanical properties of the final product are greatly impacted by factors such as density, infill type, printing orientation, layer height, and number of outline perimeters in the FDM additive manufacturing process. In addition, the interplay of these parameters significantly affects the mechanical properties [37].

In this work, used a multi-material Smart one plus model (4DS brand made by adroitec, India) FDM 3D printer called “Robust Enough” for print the test specimen with a 1.75 mm diameter ABS filament. It has a dimension of 300 × 620 × 1075 mm and features a 32-bit ARM Cortex M4 processor. The print head travel speed can be adjusted from 20 mm/s to 120 mm/s. The Simplify 3D program specifies, controls, and slices the printing parameters. Table 1 shows the ABS polymer's printing characteristics and mechanical properties. Based on existing literature [7,16], optimized printing process parameters (refer to Table 2) were selected to print the test sample for the present study.

**Table 1:** Printing characteristics and Mechanical properties of the ABS.

Properties	ABS
Filament diameter	1.75 mm
Material color	Gray
Density	1.05 g/cm^3^
Extrusion temperature^18^	220–260 °C
Bed platform temperature^18^	90–110 °C
Tensile strength^18^	43 MPa
Flexural strength^18^	66 MPa
Izod impact strength^18^	19 kJ/m^2^
Modulus of elasticity^6^	2.3 GPa
Recyclability^18^	Yes

**Table 2:** Fixed process parameters for FFF in 3D printing.

Printing parameters	Value
Nozzle diameter	0.4 mm
Initial layer height	0.27 mm
Line width	0.35 mm
Wall line width	0.35 mm
Outer wall line width	0.35 mm
Inner wall line width	0.30 mm
Top/Bottom line width	0.35 mm
Infill line width	0.4 mm
Wall thickness	1 mm
Wall line count	3
Top/Bottom thickness	1.2 mm
Printing temperature	200 °C
Build plate temperature	55 °C
Print speed	60 mm/s
Filament Flow	100%
Enable retraction	Yes
Travel speed	80 mm/s
Raster orientation	[0°]
Printing orientation	Flat [y-z]
Enable cooling	Yes

After configuring the printing process parameters on the ‘Robust Enough’ 3D printer, a Simplify 3D sliced the digital 3D model and formed a layer of extrusion road pathways. Before putting ABS filament into the FDM printer, the build plate was heated at 55 °C and printed. The nozzle temperature of 200 °C was set for the FDM process for ABS. The average printing times for the tensile and impact specimens were 90 min and 30 min, respectively, as per their specimen size with the same printing speed.

### 2.1 Design and manufacturing of samples

**Fig. 2:**
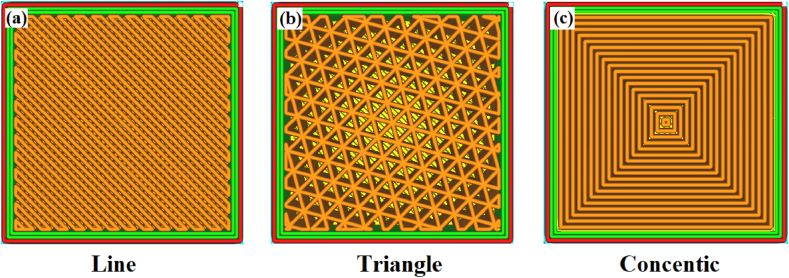
Geometrical arrangement of infill patterns (a) Line; (b) Triangle; (c) Concentric.

**Fig. 3:**
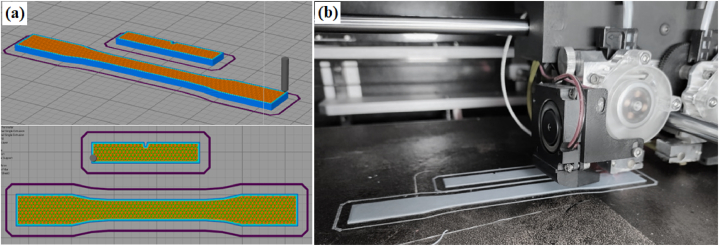
(a) Simulation of specimen on Simplify; (b) 3D Printing of specimen by 3D printer.

In the CREO parametric solid modeling software, the impact and tensile specimens of 3D models were created as per the ASTM D638 and ASTM D256 standards, respectively. In the current work, the process parameters (refer to Table 3), i.e., infill patterns, infill densities, and layer thicknesses, were varied at three levels for the fabrication sample. Nine specimens were printed for evaluation tensile and impact strength. These nine specimens (18 in total for both tests) chosen using Taguchi's L_9_ Array design of experiments, as seen in Table 4, based on varying combinations of infill density, infill pattern, and layer thickness. The specimens were fabricated using the FFF technique according to the appropriate process parameters. The geometrical structure of the line, triangle, and concentric infill patterns is presented as shown in Fig. 2 (a), (b) and (c) respectively. Fig. 3 (a) depicts a simulation of a specimen on ‘Simplify’ software prior to printing, and Fig. 3 (b) shows a specimen being printed on a 3D printer.

**Table 3:** Range of process parameters used for fabrication of sample.

Symbol	Process Parameter	Unit	Levels
A	Infill pattern	–	Line, Triangle, Concentric
B	Infill density	%	75, 80, 85
C	Layer thickness	μm	100, 200, 300

**Table 4:** L9 experiment design.

Sample No.	Control factors		
	A	B	C
S1	Line	75	100
S2	Line	80	200
S3	Line	85	300
S4	Triangle	75	200
S5	Triangle	80	300
S6	Triangle	85	100
S7	Concentric	75	300
S8	Concentric	80	100
S9	Concentric	85	200

### 2.2 Experimental set-up

The specimens with combinations of different infill patterns, infill densities, and layer thickness were made to subjected to tensile and impact testing to evaluate the consequence of printing process parameters on mechanical properties.

#### 2.2.1 Tensile strength test

**Fig. 4:**
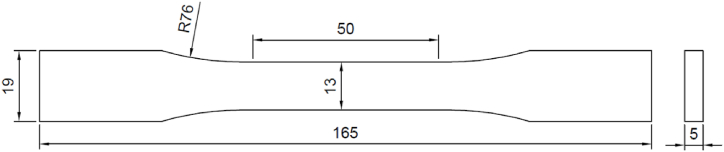
Dimensions of tensile test sample as per ASTM D638.

**Fig. 5:**
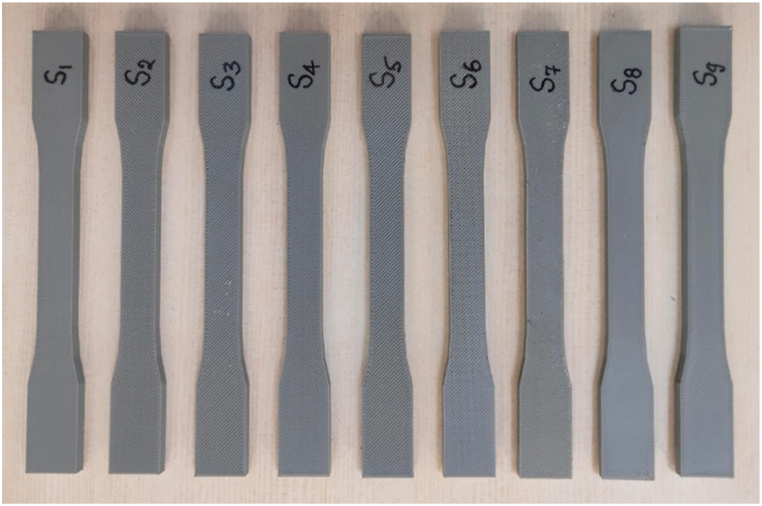
Fabricated tensile test samples.

**Fig. 6:**
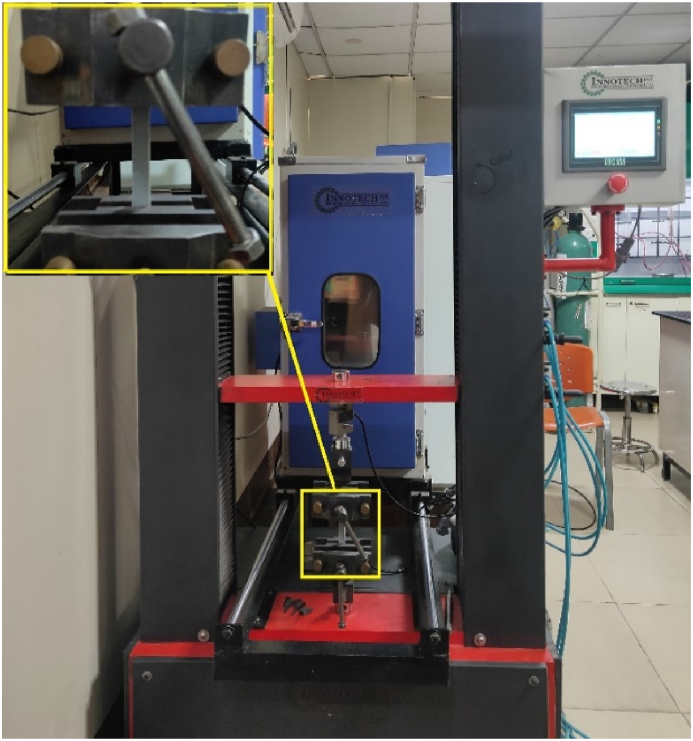
Schematic of tensile testing equipment (Universal testing machine).

For the tensile strength test, specimens were prepared as per ASTM D638 Type-I with dimensions of 50 mm gauge length, 13 mm gauge width, and 5 mm thick, as shown in Fig. 4. Fig. 5 displays the 3D printed samples used in the tensile testing. The specimens' tensile strength was measured using a computerized Universal Testing Machine (Fig. 6) (Sharda University, UP, India) with a capacity of 3 tons (model AMT-10 b y Innotech Engineering Devices Pvt. Ltd.) comes equipped with an environmental chamber capable of controlling temperature between −70 and 180 degree Celsius with an accuracy of ± 1 °C. The specimen was positioned in the jaws of the testing machine, with a grip distance of 115 mm, in accordance with ASTM D638. The test was performed at a cross-head speed of 2 mm/min. This experimentation collected % elongation, ultimate tensile strength, and yield strength at the break.

#### 2.2.2 Impact strength test

**Fig. 7:**
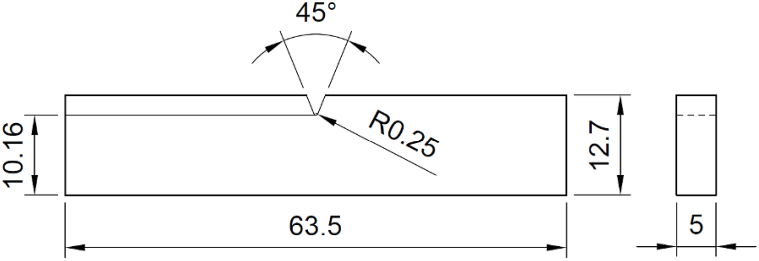
Dimensions of impact test sample as per ASTM D256.

**Fig. 8:**
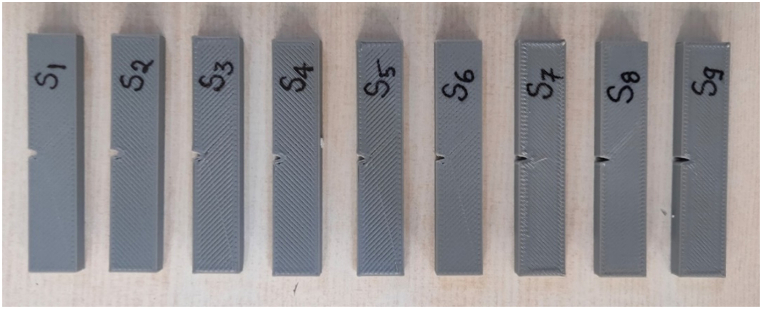
Fabricated impact test samples.

**Fig. 9:**
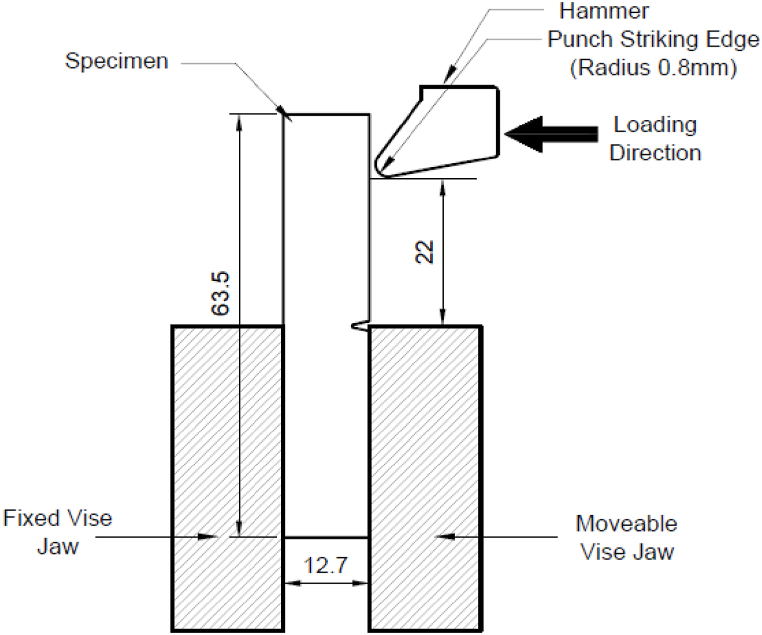
Schematic of impact testing equipment.

For the impact strength test, specimens were created in accordance with ASTM D256 with dimensions of thickness 5 mm, width 12.7 mm, length 63.5 mm, and a central notch at 45°, as shown in Fig. 7. The prepared 3D printed Izod test samples are shown in Fig. 8. A 150 J impact tester machine was used to carry out the Izod impact test. The specimens for the impact test were secured vertically in the fixture with a notched edge facing the striking edge of the pendulum. A schematic representation of this setup can be seen in Fig. 9. The pendulum was released and allowed to impact the specimen. The impact strength was estimated by the ratio of the energy absorbed before fracture (kJ) and the cross-section area of the specimen (m^2^).

## 3 Results and discussion

### 3.1 Influence of process parameter on tensile strength

The fused filament fabrication printing can cause thermal and strain hardening to occur during material extrusion, which in turn, can alter the molecular structure of ABS, leading to a strengthened material [33]. The current study examines the combined influence of three control parameters, infill pattern, densities, and layer thickness, on the tensile strength of 3D-printed ABS material.

**Fig. 10:**
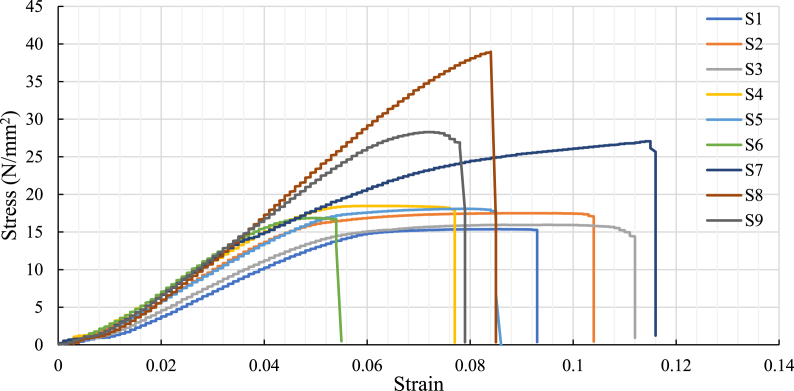
Stress-strain curves during tensile testing for all the printed specimen.

**Fig. 11:**
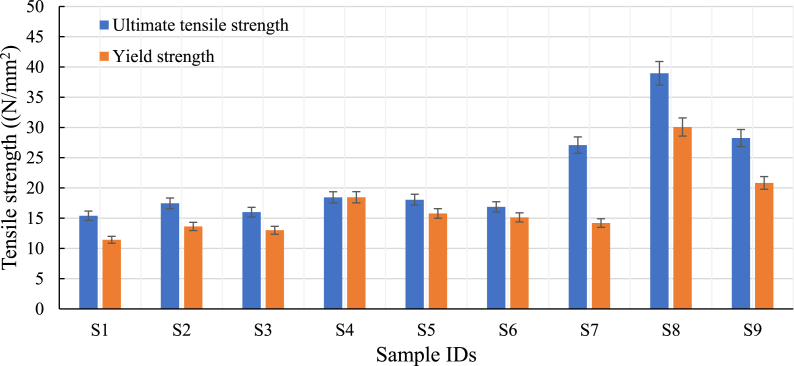
Influence of process parameter on tensile strength.

The experimental tensile strength test results (refer to Table 5) under a different set of control factors such as infill patterns (line, triangle, and concentric), infill density range (75%, 80%, and 85%), and layer thickness range (100 μm, 200 μm, and 300 μm) are shown Fig. 10. The ultimate tensile strength of the samples with a concentric infill pattern is found to be higher than those with line and triangle infill patterns for all infill densities and layer thicknesses. This result can be seen in Fig. 10, Fig. 11. The concentric patterns with 80% infill density and 100 μm layer thickness have better tensile strength than the other two infill densities (75% and 85%) and layer thicknesses (200 μm and 300 μm) for the same pattern. It was also observed that the triangle pattern showed higher UTS than the line pattern. At 80% infill, the concentric pattern (S8) had 123% and 115% higher ultimate tensile strength than line (S2) and triangle (S5) patterns respectively. The results of the study suggest that specimens with a higher infill density of 80% showed stronger bonding between layers and better resistance to deformation due to a reduction in air gaps. This may led to improved inter-layer adhesion and stronger bonding between consecutive layers by optimizing the printing parameters (such as infill density, layer thickness, and infill pattern along with printing speed and print bed temperature) resulting in increased mechanical strength, which can lead to better structural integrity, resistance to deformation, and overall performance of the material or structure [7].

**Table 5:** Tensile strength of samples.

Sample IDs	Control factors			Ultimate tensile strength (N/mm^2^)	Yield strength (N/mm^2^)	% Elongation
	A	B	C			
S1	Line	75	100	15.40	11.43	9.29
S2	Line	80	200	17.46	13.63	10.39
S3	Line	85	300	15.99	13.00	13.12
S4	Triangle	75	200	18.44	18.46	7.65
S5	Triangle	80	300	18.05	15.78	8.53
S6	Triangle	85	100	16.87	15.13	5.44
S7	Concentric	75	300	27.08	14.20	15.66
S8	Concentric	80	100	38.95	30.07	8.46
S9	Concentric	85	200	28.25	20.83	7.87

However, the % of elongation at the breakpoint was reduced to 8.46. At 75% infill density along with layer thickness (100 μm), the line (S1) pattern had the lowest tensile strength compared to triangle (S4) and concentric (S7) patterns. However, the 85% infill concentric had a yield strength of 20.83 MPa, while the line and triangle had a yield strength of 13.0 MPa and 15.13 MPa, respectively. The concentric pattern (S8), having 80% infill density and 100 μm layer thickness, is the strongest among others and has an ultimate tensile strength of 38.95 MPa. The printing with smaller layer thickness can lead to higher resolution and finer details, but it can also result in increased surface exposure, which may cause degradation or weakening of the material [18]. Therefore, the best outcome is achieved through a minimum layer thickness combined with high infill density, leading to improved layer bonding strength. The line pattern (S1) with 75% infill density and 100 μm layer thickness is the weakest having an UTS of 15.40 MPa.

**Fig. 12:**
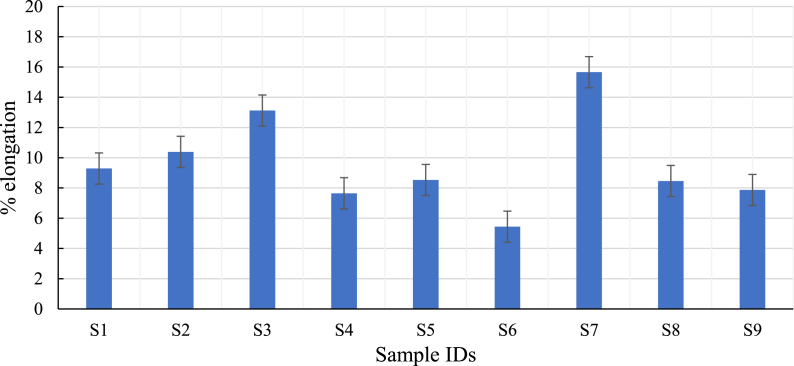
Influence of process parameter on % elongation.

The elongation percentage at the breakpoint of the concentric pattern (S7) was higher than other concentric patterns (S8 and S9) and all lines (S1, S2, and S3), and triangle (S4, S5, and S6) patterns. The experimental result showed (refer to Fig. 12) that the concentric pattern with a higher elongation at break percentage has higher ductility. The result reveals that the concentric pattern was more likely to deform before fracturing under tensile load than other line and triangle patterns. Therefore, it shows that every process parameter has a vital role that affects the properties of test samples individually.

### 3.2 Influence of process parameter on impact strength

**Fig. 13:**
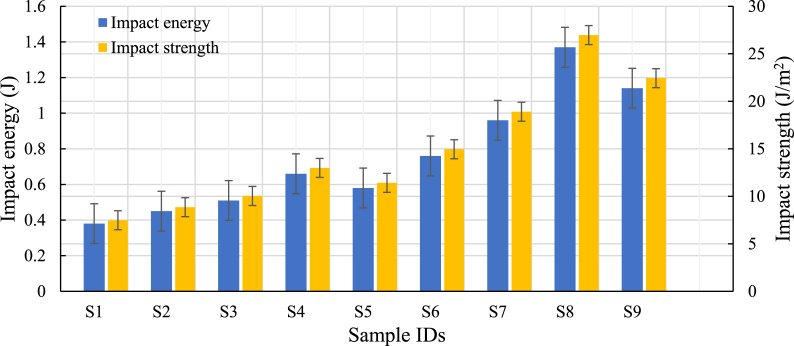
Influence of process parameter on impact strength.

The impact strength of test samples was conducted to estimate the capability to absorb energy during the plastic deformation of the ABS (Acrylonitrile Butadiene Styrene) 3D printed components in the concentric patterns, triangle, and combination of line and along with infill densities (75%, 80%, and 85%) and layer thickness (100, 200, and 300 μm). The impact test results of samples for different combinations are recorded and mentioned in Table 6. The result showed that the concentric patterns show better impact-resisting performance (refer to Fig. 13) than line and triangular patterns.

**Table 6:** Impact strength of samples.

Sample IDs	Control factors			Impact energy (J)	Impact strength (kJ/m^2^)
	A	B	C		
S1	Line	75	100	0.38	7.48
S2	Line	80	200	0.45	8.86
S3	Line	85	300	0.51	10.04
S4	Triangle	75	200	0.66	12.99
S5	Triangle	80	300	0.58	11.42
S6	Triangle	85	100	0.76	14.96
S7	Concentric	75	300	0.96	18.90
S8	Concentric	80	100	1.37	26.97
S9	Concentric	85	200	1.14	22.44

The results showed that the absorption of impact energy increased with the change in infill pattern from line to triangle and concentric, as well as changes in infill density and layer thickness. The concentric pattern (S8) had 168% and 80% higher impact strength than line (S3) and triangle (S6), respectively. The crack in the concentric infill pattern did not spread continuously, which leads to increased resistance to fracture. The interruption of crack propagation due to the concentric pattern leads to an increase in the structure's energy-absorbing ability, as the part is better able to resist heavy stress and distribute the stress more evenly. Thus, the results show improved the tensile strength as well as impact strength compared to other infill patterns. As the percentage of infill density increases from 75% to 85%, the energy absorbed by samples grows for line and triangle patterns. However, the rate decreases beyond 80% for the concentric pattern (S8), as in Fig. 13. The impact strength is also affected by the thickness of the printed specimen's layers. With decreasing layer thickness, the amount of deposited material decreases, making it possible for residual heat during subsequent layer deposition to enhance polymer entanglement, leading to improved material strength.

With the specimen's impact-absorbing capabilities in mind, the impact resistance of each combination of layer thickness, density, and infill pattern was carefully analyzed. The impact energy of the concentric pattern was found more significant for all infill densities and layer thicknesses when compared to the line and triangle infill patterns (Fig. 13). The concentric patterns with 80% infill density and 100 μm layer thickness have more excellent impact-resistant capability than the other two infill densities (75% and 85%) for the same pattern. The triangle pattern has a higher impact-resistant ability than the line pattern. Therefore, decreasing layer thickness along with increased infill density enhanced combined adhesion bonding strength between the layers as a result of increased polymer chain entanglement from the increased number of deposition interfaces and is also dependent on residual heat between previously printed layers and newly deposited material.

## 4 Conclusion

The impact strength and ultimate tensile strength of 3D printed ABS materials manufactured using the FFF technique were examined. The following conclusions are taken from the results and discussion.

1. Concentric infill pattern had the most influential process parameter because this pattern leads to an increase in resistance to fracture and improved tensile strength and impact strength due to the interruption of crack propagation and better stress distribution.
2. The loading capacity and mechanical strength, such as tensile yield, ultimate tensile strength, and elastic modulus, of a printed sample are influenced by the number of layers deposited. An increase in the number of layers within a fixed volume leads to a corresponding rise in the overall strength of the sample.
3. The ultimate tensile strength of the concentric pattern was found to be 123% and 115% higher than the line and triangle infill patterns.
4. The concentric patterns with 80% infill density and 100 μm layer thickness, enhances the mechanical strength of the part by promoting inter-layer bonding between consecutive layers. The results have excellent tensile strength than the other two infill densities (75% and 85%) and (200 μm and 300 μm) for the same pattern.
5. The concentric pattern also demonstrated a higher elongation at break, which is more likely to deform before fracturing under tensile load than other line and triangle patterns.
6. It has also been noticed that impact strength is mostly determined by the type of mesostructure (infill pattern), infill density, and layer thickness. The concentric infill pattern with 80% density and 100 μm layer thickness demonstrated the 168% and 80% highest energy absorbing potential across the line and triangle patterns, respectively, with each infill density range (from 75 to 85%) and layer thickness range (from 100 to 300 μm).
7. The impact strength of line pattern with 75% density and 100 μm layer thickness had been observed lower among all samples under impact loading.

## Author contribution statement

Anant Prakash Agrawal: Conceived and designed the experiments; Performed the experiments; Analyzed and interpreted the data; Contributed reagents, materials, analysis tools or data; Wrote the paper.

Virendra Kumar, Prabhu Paramasivam: Analyzed and interpreted the data; Contributed reagents, materials, analysis tools or data; Wrote the paper.

Jitendra Kumar: Conceived and designed the experiments; Contributed reagents, materials, analysis tools or data; Wrote the paper.

Seshathiri Dhanasekaran, Lalta Prasad: Contributed reagents, materials, analysis tools or data; Wrote the paper.

## Data availability statement

Data will be made available on request.

## Declaration of competing interest

The authors declare that they have no known competing financial interests or personal relationships that could have appeared to influence the work reported in this paper
